# Increased incidence of invasive bacterial disease in chronic obstructive pulmonary disease compared to the general population-a population based cohort study

**DOI:** 10.1186/1471-2334-14-163

**Published:** 2014-03-25

**Authors:** Malin Inghammar, Gunnar Engström, Bengt Ljungberg, Claes-Göran Löfdahl, Adam Roth, Arne Egesten

**Affiliations:** 1 Infection Medicine, Department of Clinical Sciences Lund, Lund University, Skåne University Hospital, Lund, Sweden; 2 Respiratory Medicine & Allergology, Department of Clinical Sciences Lund, Lund University, Skåne University Hospital, Lund, Sweden; 3 Cardiovascular Epidemiology Research Group, Department of Clinical Sciences Malmö, Lund University, Skåne University Hospital, Malmö, Sweden; 4 Medical Microbiology, Department of Laboratory Sciences Malmö, Lund University, Skåne University Hospital Malmö, Malmö, Sweden; 5 Department of Infectious Diseases, Skåne University Hospital, SE-221 85 Lund, Sweden

**Keywords:** Bacteraemia, Epidemiology, Chronic obstructive pulmonary disease, Infection, Sepsis

## Abstract

**Background:**

Innate defence mechanisms of the airways are impaired in chronic obstructive pulmonary disease (COPD), predisposing patients to lower respiratory tract infections, but less is known about the association with other infections. In this population-based cohort study, we investigated the associations between COPD and invasive bacterial disease by comparing incidence rates of bacteraemia in COPD patients and randomly selected reference individuals from the general population.

**Methods:**

In this population based cohort study all patients with COPD, ≥40 years of age, who were discharged from hospitals in southern Sweden between 1990 and 2003 were identified in the Swedish Inpatient Register (n = 15,403). Age and gender matched reference individuals were randomly selected from the general population. Records were cross-referenced to the microbiological databases covering the region, 1990–2010. The hazard ratios (HR) of bloodstream infections and hospitalisations for infections were estimated by Cox proportional hazards regression.

**Results:**

We found that individuals with COPD had a 2.5-fold increased incidence of bacteraemia compared to the reference individuals from the general population adjusted for other co-morbidity and socio-economic status (hazard ratio: 2.5, 95% confidence interval: 2.2-2.7). The increased incidence of bacteraemia was paralleled by an increased incidence of hospitalisation for non-respiratory infections, i.e., skin infections, pyelonephritis, or septic arthritis. Despite higher absolute rates of bloodstream infections among COPD patients than among the general population, the distribution of different pathogens was similar.

**Conclusions:**

In summary this population-based study shows COPD is associated with an increased incidence of invasive bacterial infections compared to the general population, indicating a general frailty of acquiring severe infections in addition to the specific susceptibility to infections of respiratory origin. The underlying contributory factors (e.g. smoking, corticosteroid use, co-morbid diseases or a frailty inherent to COPD itself) need to be disentangled in further studies.

## Background

The prevalence of chronic obstructive pulmonary disease (COPD) is increasing worldwide, and it is estimated that COPD will become the third leading cause of death in 2020 [1]. In Sweden, approximately 500,000 individuals out of a population of 9 million suffer from COPD [2], and approximately 2,500 die from the disease annually [3].

The healthy lung has a range of defence mechanisms that protect the lower respiratory airways against invading microbial pathogens. In COPD, several of these mechanisms are impaired [4], which predisposes patients to both acute and chronic lower respiratory tract infections [5]. While the association between COPD and respiratory infections has been extensively studied, less is known about the associations between COPD and other types of severe infections.

COPD is a well known risk factor for invasive disease from *Streptocccus pneumoniae* and has been suggested to be associated with invasive disease from *Pseudomonas aeruginosa*[6,7]. Whether the incidence of invasive bacterial infections from other pathogens associated with COPD, e.g., *Haemophilus influenzae,* is also increased or whether COPD patients have a general susceptibility to invasive bacterial infections is not known.

In the present population-based cohort study, COPD patients and randomly selected reference individuals from the general population living in southern Sweden, an area with approximately 1.2 million inhabitants, were compared to investigate whether underlying COPD affected the risk of acquiring invasive bacterial infections, the pattern of bacterial pathogens or the spectrum of severe infections.

## Methods

### Setting

Swedish health care is publicly financed, and all inpatient care is provided independently of health insurance and the patient’s financial status.

### Case retrieval and control selection

All individuals ≥ 40 years of age living in the county of Skåne with a hospital discharge diagnosis of COPD according to the *International Classification of Diseases* (Ninth version: 491–492, 496; Tenth version: J41-J44) 1990–2003, either as a main or additional diagnosis, were identified in the Swedish Inpatient Registry. These will be referred to as “COPD-patients”. For each individual, Statistics Sweden randomly selected one reference individual from the general population in the Total Population Registry matched for gender, year of birth, and county of residence at 31 December of the preceding year of the first hospital discharge listing a COPD diagnosis. A unique, lifelong ten-digit personal identification number assigned to each person living in Sweden provides the possibility of cross-referencing in national databases.

All COPD-patients and general population controls were linked to national databases to obtain information on vital status, date of emigration, and socio-economic status. Information on co-morbidity was obtained through linkage with the national Inpatient Registry and the Cancer Registry. Co-morbidity was defined as a prior hospital discharge diagnosis of diabetes mellitus, kidney failure, cardiac disease, alcohol-related disease, liver failure, connective tissue disease, immune-deficiency, total number of hospitalizations and total duration of hospital stay before inclusion. In addition a diagnosis of haematological or solid cancer in the Cancer Registry up to five years before inclusion was noted (see Appendix for specific ICD-codes). The study was approved by the Lund University Research Ethics Committee (590/2004 and 270/2012).

### Follow-Up

The records were cross-referenced to the databases of all microbiological laboratories in the region to obtain information on all blood cultures with growth. Follow-up started 30 days after the first hospitalization with COPD and on the corresponding date for the reference individuals and ended on the date of emigration, date of death or at 31 December 2010, whichever came first. Individuals were not considered at risk less than 30 days after an episode for all outcomes except endocarditis and septic arthritis were a lag time of 60 days was used due to long treatments. In addition, information on all hospital discharge diagnoses between 1990 and 2010 was obtained.

### Statistical analysis

The incidence rates for pooled bloodstream infections, infections with specific bacterial species and hospitalization for infections during follow-up were calculated. Relative ratios for the association with COPD and *first* subsequent infection were estimated using Cox proportional hazards models. All models were internally stratified by year of birth, age and gender, using time since inclusion as the timescale, and were subsequently adjusted for socio-economic status and co-morbidity at baseline. Interactions between COPD status and age at inclusion, gender, and prior hospitalization on outcome were tested by entering interaction terms in the fully adjusted Cox models. Proportionalities of hazards were assessed graphically and by the method using Schoenfeld residuals [8]. All analyses were performed using STATA/SE (version 10.1 for Windows; StataCorp LP, College Station, USA).

## Results

### Study population and exclusions

A total of 17,955 individuals with a discharge diagnosis of COPD were identified. Among these, 54 (0.3%) individuals were excluded because of data irregularities, and 1,754 (9.8%) individuals who died before the start of follow-up (<30 days after the date of inclusion) were also excluded.

Because reference individuals were randomly selected from the background population, some of these also appeared as COPD patients during follow-up. Among the reference individuals, we excluded 449 (2.5%) who died before the start of follow-up and 362 (2.0%) who had been hospitalised with a COPD diagnosis prior to inclusion. An additional 930 (5.2%) reference individuals were included in the analysis but censored by the date of the first hospitalization listing COPD.

Due to the matched design 744 (4.1%) COPD patients lacking controls due to exclusions and 1,741 (9.7%) controls were excluded. Analysis was based on the remaining 15,403 case–control pairs. Baseline characteristics of the study populations are shown in Table 1.

**Table 1:** Demographic characteristics of the study population

	***COPD patients n = 15,403 (100%)***	***Reference individals***^***1***^ ***n = 15,403 (100%)***	***p-value***
*Age distribution (years)*			*NA*
*40-59*	1,858 (12.1%)	1,858 (12.1%)	
*60-79*	9,346 (60.7%)	9,346 (60.7%)	
*≥80*	4,199 (27.3%)	4,199 (27.3%)	
*Gender*			*NA*
Male	8,265 (53.7%)	8,265 (53.7%)	
*Socio-economic status*			<0.01
Non-manual	2,569 (16.7)	3,684 (23.9)	
Manual	4,473 (29.0)	4,002 (26.0)	
Other	1,646 (10.7)	1,767 (11.5)	
Outside workforce ^2^	6,715 (43.6)	5,950 (38.6)	
*Co-morbidity*^3^			
Diabetes mellitus	966 (6.3)	557 (3.6)	<0.01
Kidney failure	107 (0.7)	42 (0.3)	<0.01
Cardiac disease	3,775 (24.5)	1,993 (12.9)	<0.01
Alcohol-related disease	573 (3.7)	159 (1.0)	<0.01
Liver failure	96 (0.6)	59 (0.4)	<0.01
Connective tissue disease	428 (2.8)	210 (1.4)	<0.01
Immunodeficiency	57 (0.4)	23 (0.2)	<0.01
HIV	8 (0.05)	2 (0.01)	0.06
Solid cancer	1,322 (8.6)	759 (4.9)	<0.01
Hematological cancer	110 (0.7)	42 (0.3)	<0.01
Total days in hospital (mean/median)	37.7 / 12	22.5 / 3	<0.01
No. of discharges, all diagnoses	4.4 / 2	2.2 / 1	<0.01

^1**−**^Control subjects were matched for year of birth, gender and county of residence by the 31^st^ of December of the year before first hospital discharge listing a COPD diagnosis; ^2−^Subjects with missing information on socio-economic status were mainly older pensioners, unemployed, disability pensioners, homemakers and students: ^3^-Comorbidity was based on discharge codes in the Inpatient Register before inclusion; see Appendix for specific ICD codes.

### Bloodstream infections

In the 30,806 cases and controls, a total of 2,137 episodes of bloodstream infections occurred (1,265 episodes in 1,105 COPD patients and 872 episodes in 759 reference individuals) during 87,258 and 140,063 person-years of follow-up, respectively. Bacterial species of commensal skin flora (e.g., coagulase-negative staphylococci, *Corynebacteriaceae* and *Propionibacteriaceae*) were considered as contaminants and not included.

**Figure 1:**
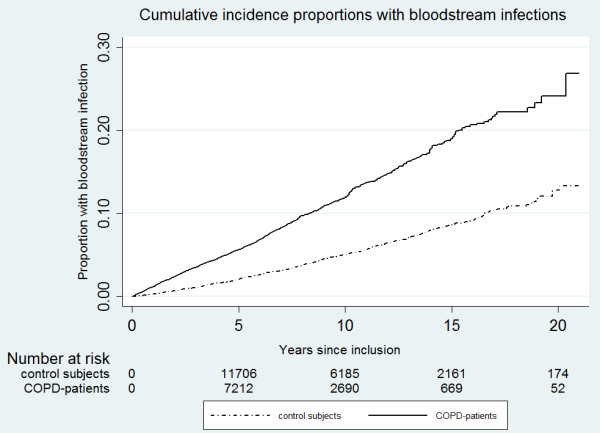
**Cumulative incidence proportions for bloodstream infections for patients hospitalised with a COPD diagnosis and control subjects from the general population.** Individuals were censored on the first episode of bloodstream infection.

The resulting crude incidence rates were 145.0 /10,000 person-years of follow-up (95% confidence interval [95% CI] 137.2 to 153.2) in COPD patients and 62.3 (95% CI 58.3 to 66.5) in reference individuals, (see Table 2). Median-time to first episode was 4.3 years in COPD-patients and 6.7 years in reference individuals, the cumulative incidences are shown in Figure 1. This corresponds to a 2.5-fold increased risk of time to first episode as estimated by Cox regression (stratified by age, gender and year of birth and adjusted for co-morbidity and socio-economic status), as outlined in Table 3.

**Table 2:** Age-specific incidence rates of bacteremia for individuals previously discharged with a COPD diagnosis and reference individuals from the general population

***COPD-patients***			***Control Subjects***		
**Pathogen**	**N (%)**^**1**^	**Incidence**^**2**^	**N (%)**^**1**^	**Incidence**^**2**^	**HR (95% CI)**^**3**^
Pooled bloodstream infections	1,265(100)	145.0 (137.2-153.2)	872 (100)	62.3 (58.3-66.5)	2.5 (2.2-2.7)
40-59 years	157 (12.4)	87.4 (74.8-102.2)	68 (7.8)	29.1 (22.9-36.9)	3.1 (2.2-4.3)
60-79 years	850 (67.2)	154.0 (144.0-164.7)	573 (65.7)	61.5 (56.7-66.8)	2.6 (2.3-3.0)
≥80 years	258 (20.4)	183.0 (162.0-206.8)	231 (26.5)	98.2 (86.3-111.7)	1.9 (1.5-2.3)
*Staphylococcus aureus*	183 (13.5)	21.0 (18.1-24.2)	127(12.8)	9.1 (7.7-10.8)	2.4(1.8-2.9)
*Streptococcus pneumoniae*	157 (11.2)	18.0 (15.4-21.0)	60(6.1)	4.3 (3.3-5.5)	4.1 (3.0-5.7)
*Pseudomonas aeruginosa*	45 (3.2)	5.2 (3.9-6.9)	26 (3.0)	1.9 (1.3-2.7)	3.0 (1.8-5.2)
*Enterobacteriacea*^*4*^	985 (42.0)	112.9 (106.0-120.2)	786 (48.5)	56.2 (52.3-60.2)	2.1 (1.9-2.5)
*Enterococci*	94 (7.2)	10.8 (8.8-13.1)	74 (8.2)	5.3 (4.2-6.6)	1.9 (1.3-2.6)
*Streptococcus pyogenes*	22 (1.6)	2.5 (1.7-3.8)	15 (1.6)	1.1 (0.6-1.8)	2.8 (1.4-5.6)^5^
*Haemophilus influenzae*	10 (0.7)	1.1 (0.6-2.1)	7 (0.7)	0.5 (0.2-1-0)	2.8 (1.0-7.7)^5^
*Moraxella catarrhalis*	1	NA	0	NA	NA

^1^-Percentage of total blood cultures with growth in either COPD patients or control subjects. ^2^- Total (crude) incidence of bloodstream infections per 10,000 person-years of follow-up. Infectious episodes were separated by <30 days.^3^-Hazard ratio of time to *first* bloodstream infection estimated by Cox regression using time since inclusion as the time scale and stratified for age, year of birth and gender and adjusted for socio-economic position and co-morbidity conferring increased risk of severe bacterial infections. ^4^-*E. coli, Citrobacter spp., Enterobacter spp., Klebsiella spp., Providencia spp., Proteus spp., Serratia marcescens*.; ^5^-Due to the limited number of outcomes, this Cox model was only adjusted for total days spent in the hospital prior to inclusion.

**Table 3:** Hazard ratios for bacteraemia for underlying medical conditions, total use of inpatient care and socio-economic position before inclusion

	**Univariate HR (95% CI)**^**1**^	**Multivariate HR (95% CI)**^**2**^
COPD	2.8 (2.5- 3.0)	2.5 (2.2-2.7)
Diabetes mellitus	2.4 (2.0-2.8)	1.7 (1.4-2.0)
Kidney failure	7.7 (5.0-11.8)	4.7 (3.0-7.5)
Cardiac disease^3^	1.7 (1.5-1.9)	1.2 (1.0-1.3)
Alcohol-related disease	2.0 (1.5-2.7)	1.2 (0.9-1.6)
Liver failure	2.7 (2.0-3.5)	2.3 (1.4-3.7)
Connective tissue disease	2.9 (1.8-4.6)	1.8 (1.4-2.4)
Immunodeficiency	3.8 (2.1-6.7)	1.8 (1.0-3.3)
Solid cancer	1.5 (1.2-1.8)	1.3 (1.1-1.6)
Hemathological cancer	5.6 (3.5-8.8)	4.7 (2.9-7.5)
Total days in hospital^4^	1.0 (1.0-1.0)	1.0 (1.0-1.0)
Total no. of discharges, all diagnoses^5^	1.6 (1.5-1.7)	1.2 (1.1-1.3)
Socio-economic position		
Non-manual	1	1
Manual	1.2 (1.1-1-4)	1.1 (0.9-1.3)
Other	1.2 (1.0-1.4)	1.1 (1.0-1.3)
Missing	1.4 (1.2-1.7)	1.3 (1.1-1-5)

^1^- Hazard ratio of time to *first* bloodstream infection, having the condition compared to not having the condition, estimated by Cox regression using time since inclusion as the time scale and stratified for age, year of birth and gender; ^2^- Adjusted for all of the variables in the column; ^3^-Ischeamic heart disease or heart failure.^4^-Per 10-days increment; ^5^-Per increment of ten hospitalizations.

The *absolute* incidence increased with age, but the *relative* risk decreased with increasing age group (p = 0.01 from likelihood ratio test for interaction) (see Table 2). There was no evidence of an interaction with gender (p = 0.5 from interaction test).

A sensitivity analysis, using an in-patient control group, restricted to those pairs of COPD patients and controls in which the reference individual had been hospitalised prior to inclusion (n = 18,148), yielded slightly more conservative estimates, adjusted HR of 2.2 (95% CI 2.0-2.5) (p = 0.01 from interaction test). The mean age in this group was higher than in those with no previous hospitalization (74.6 (SD 9.9) vs. 69.7 (SD 10.3), p = 0.01). In secondary analysis, subjects were stratified according to the total number of co-morbidities predisposing to severe infections, which also yielded similar results, adjusted HR 2.4 (95% CI 2.2-2.7).

A sensitivity analysis, treating co-morbidity covariates as time-dependent (i.e. co-morbidity status up to first episode of bacteraemia) yielded similar results, adjusted HR of 2.2 (95% CI 2.0-2.5). A sensitivity analysis excluding COPD patients with concurrent asthma discharge diagnosis showed identical results. Rerunning analyses not censoring control subjects with subsequent COPD diagnosis yielded similar results (adjusted HR: 2.3; 95% CI: 2.1-2.6).

### Distribution of pathogens

Despite higher absolute rates of bloodstream infections among COPD patients than among reference individuals, the distribution of different pathogens was similar. COPD patients had a larger proportion of *S. pneumoniae* (11.2% vs. 6.1%) and *S. aureus* (13.5% vs. 12.8%) and a corresponding smaller proportion of *Enterobacteriaceae* compared to controls subjects (42.0% vs. 48.5%). Specific incidence rates and the corresponding HRs are presented in Table 2.

### Clinical diagnosis

There was an overall increased incidence of hospitalisations due to infectious diseases among COPD patients during follow-up compared to the reference cohort, even for infections of non-respiratory origin (e.g. endocarditis, skin infections, septic arthritis and pyelonephritis), see Table 4.

**Table 4:** Incidence rates of hospitalizations for infectious diseases in the Inpatient Register for individuals previously discharged with a COPD diagnosis and control subjects from the general population

	***COPD***		***Control subjects***		
**Type of infection**	**N**	**Incidence**^**1**^	**N**	**Incidence**^**1**^	**HR**^**2**^
Pnemonia	8,581	983 (963–1,004)	2,638	188 (181–195)	4.7 (4.4-4.9)
Skin infection	698	80 (74–86)	331	24 (21–26)	3.3 (2.8-3.8)
CNS infection	18	2.0 (1.3-3.2)	23	1.6 (1.1-2.4)	1.1 (0.6-2.3)^3^
Endocarditis	60	6.9 (5.3-8.9)	34	2.4 (1.7-3.4)	2.8 (1.7-4.7)
Pyelonephritis	500	57 (52–62)	300	22 (20–24)	2.4 (2.0-2.9)
Septic arthritis	93	10.7 (9.7-13.1)	80	5.7 (4.6-7.1)	1.9 (1.3-2.6)

^1^- Crude incidence of hospitalizations for infectious diseases per 10,000 person-years of follow-up. Infectious episodes were separated by >30 days for pneumonia, skin infection, CNS infection and pyelonephritis and >60 days for endocarditis and septic arthritis. ^2^-Hazard ratio of time to first hospitalization for infection estimated by Cox regression using time since inclusion as the time scale and stratified for age, year of birth and gender and adjusted for socio-economic status and co-morbidity. ^3^-Due to the limited number of outcomes, this Cox model was only adjusted for any co-morbidity (yes/no) prior to inclusion.

## Discussion

In this population-based study of 15,000 COPD patients, we show that individuals discharged with a COPD diagnosis have a 2.5-fold increased incidence of invasive disease from a wide range of bacterial pathogens compared to the general population, independently of socio-economic status and co-morbidities at base-line.

We hypothesized an increased incidence of invasive diseases caused by bacteria associated with colonisation of the airways in COPD, due to impaired barrier functions. But increased incidence was also observed for other major pathogens normally associated with non-respiratory infections, i.e., *Staphylococcus aureus* and enterobacteriaceae, indicating a general susceptibility to invasive infections of non-respiratory origin, which in turn could be due to different mediating factors.

COPD is currently recognised as a disease with systemic manifestations not limited to airflow obstruction and is associated with several serious co-morbidities [9]. It is therefore plausible to believe that COPD also confers increased incidence of invasive bacterial infections of non-respiratory origin. In contrast to our results, a Danish study concluded that underlying COPD did not predict hospitalizations due to infections outside the respiratory system [10]. The study was based on 2,200 individuals with COPD who were subdivided into GOLD classes according to FEV_1_. Re-examining the incidence rates in the Danish study does suggest an increased risk of hospitalisation for non-respiratory infectious diseases for COPD as a group, although there was no clear trend of increased risk with increasing GOLD stage. This classification, based solely on FEV_1_, may not be sufficient to capture the full effect of co-morbidities in COPD [11].

The increased incidence of infection in COPD could also, to some extent, be mediated by the use of corticosteroids, anticholinergic inhalants or smoking. A limitation of the present study is the lack of individual information on COPD severity, corticosteroid use or smoking status which is not available in the national databases.

The use of corticosteroids is an indisputable risk factor for infection. However, risk estimates from observational studies are limited by confounding by indication and the nature of the underlying disease is an important modifier of the infection risk, making it difficult to generalise results from different disease groups [12]. In a pooled analysis of 71 controlled clinical trials, a relative risk of 1.6 for infectious complications were reported in patients given more than a cumulative dose of >700 mg prednisone [12], but a stratified analysis of trials including patients with pulmonary diseases showed no increased risk of infectious complications [12]. Neither Smyllie et al. (case–control study of 500 patients with respiratory diseases) [13] nor Niewoehner et al. (randomised controlled trial of oral steroid treatment for two or eight weeks in COPD), found statistical significant differences in secondary infections [14]. In contrast, some meta-analyses of randomised control trials have reported that treatment with inhaled corticosteroids is associated with a 50-70% increased risk of pneumonia [15,16]. However, no increased risk was reported in pooled analyses of trials using budesonide [17]. A side-effects of anticholinergic agents is urinary retention, which can predispose to urinary tract infections. Barr et al*.* (systematic meta-analysis of 3 clinical trials) report an OR of 1.6 for urinary tract infections in users of tiotropium vs. placebo [18].

Smokers have an increased risk of infectious complications but it is has not been shown that smoking is a risk factor for bacteraemia not mediated through bacterial colonization of the airways [19,20]. The comparisons in the present study are not made between a smoking and a non-smoking group; population-based studies of COPD in Sweden have reported current smoking rates from 47% (in 1992) to 34% (in 2004) in COPD patients compared to 33% and 13%, respectively, in non-COPD subjects in these studies [21,22]. A survey of COPD patients in secondary care (2007) showed that 23% were still smokers [23] compared to national estimates of around 16% (2005) in the adult population (>45 years of age) [24].

Register-based studies from national databases have several advantages but also important limitations. The study design, using a reference population that is randomly drawn from the background population and standardised for age, gender, county, allows adjustments for co-morbidity confounders, which is not possible in designs based on national estimates, producing SIRs (standardised incidence ratios).

The study covers all patients hospitalised with COPD in Skåne during the observation period, but individuals with COPD treated only as outpatients were not included in the analysis. However, we found no effect modification by the level of COPD diagnosis; thus risk estimates were similar in subjects identified with COPD as main diagnosis (hospitalised *because* of COPD) and as an additional one (hospitalised *with* COPD). The prevalence of physician-diagnosed COPD in Sweden is estimated to be 5-10%, and in our study, 7.2% of the randomly selected general population control subjects had a hospital discharge diagnosis of COPD during the observation period. This result implies that a large proportion of patients with COPD will eventually be hospitalised either because or with COPD.

General validation studies of the Swedish Inpatient Registry indicate that the coverage is above 98% and that almost 90% of the reported diagnoses are correct [25-27]. We have previously validated the COPD in this registry [28]. The amount of evidence supporting the diagnosis varied, but less than 10% were considered to be misclassified or having an uncertain COPD diagnosis. All stages of COPD-severity were seen since patients were admitted due to a number of reasons, not necessarily linked to COPD, but the degree of validity did not differ between COPD as a main or additional diagnosis. A misclassification of the COPD-diagnosis in the present study, in addition to the inclusion of COPD patients receiving only outpatient care as control subjects, would presumably bias the estimates downwards.

Diagnoses of infectious diseases from the Swedish Inpatient Registry have not been validated separately, except for infections in intensive care (CNS-infections, pneumonia and sepsis) with the overall pattern of varying sensitivity (38-99%) but high specificity (98.6-99.6%) [29]. The misclassification of clinical diagnosis are assumed to be non-differential except for pneumonia, which could theoretically be prone to increased misclassification in COPD patients due to the sometimes diagnostic dilemma to differ between episodes of exacerbation, heart failure or pneumonia [30].

We adjusted our models for the use of inpatient care at baseline and inpatient co-morbid conditions associated with COPD and conferring increased risk of infection. Residual confounding from co-morbid conditions requiring only outpatient care cannot be ruled out.

In Sweden, there is as general recommendation to perform blood culture (at least) before treating patients with intravenous antibiotics. Whether the adherence to these guidelines is greater or less for COPD patients than for individuals without COPD is not known. In the present study the, hazard ratios were similar when using an in-patient control group, indicating that the risk of ascertainment bias is limited.

We conducted a population-based cohort study of the incidence of severe bacterial infections in COPD patients spanning 20 years covering all residents of the County of Skåne, an area with approximately 1.2 million inhabitants. We believe that the results are generalizable to many settings, although differences in population composition or access to health care are likely to influence the results.

## Conclusion

This study shows that COPD patients have an overall increased incidence of invasive bacterial infections compared to the general population, indicating a general susceptibility in addition to the specific susceptibility to invasive infections of respiratory origin. The mediating factors, smoking, corticosteroid use, co-morbid diseases or a frailty inherent to COPD itself, and whether this propensity influences the mortality rates, need to be disentangled in further studies.

### Consent

The study was approved by the Lund University Research Ethics Committee (590/2004 and 270/2012). In accordance with Swedish regulations, informed consent was not obtained.

## Appendix

Medical diagnoses in the Inpatient Registry and Cancer Registry are coded according to the International Classification of Disease, ninth and tenth revision, as follows: Liver disease (ICD-8: 570, 571,90, 571,98, 571, 99, 573; ICD-9: 571E, 571 F, 571G, 571 W, 571X, 572–573; ICD-10: K71-76), Renal failure (ICD-8: 582; ICD-9: 585–586; ICD-10: N18-19), Chronic obstructive pulmonary disease (COPD) (ICD-8: 491–492; ICD-9:491–492, 496; ICD-10: J41-J44), Heart failure: ICD-8: 427,00, 428; ICD-9: 428; ICD-10: I50, Ischemic heart disease: ICD-8: 410–414; ICD-9: 410–414; ICD-10: I20-I25), HIV (ICD-9: 279 K, V02J; ICD-10:B20-24; Immuno-deficiency (ICD-8: 288, 275,00, 275,10; ICD-9: 279 J,L,M,N,W,X; ICD-10:D70, D71, D80-84), Connective tissue disease (ICD-8: 712, 714,93, 716,734; ICD-9: 710, 714, 720, 725; ICD-10: M30-35, M05-09), Alcohol-related disease (ICD-8: 291, 303; 980, 571,00, 571, 01; ICD-9: 291, 303, 305A,357 F, 571A, 571B, 571C, 571D, 980A; ICD-10: F10, K70, E512, G312, G621, G721, K852, K860, X45, T45, Z714), Diabetes mellitus (ICD-8: 250; ICD-9: 250; ICD-10: E10-E11), Haematological cancer (ICD-7:200–204, ICD-9: 200–208; ICD-10: C81-C96, D46), Solid cancer (ICD-7: 140–199; ICD-9: 140–199, 230–234, ICD-10: C00-C81, D00-D09). Pneumonia (ICD-9:481–483, 486; ICD-10:A48.1, B01.2, J13-J16, J18). Skin infections (ICD-9:035, 040A; ICD-10:A46, A48.0). Pyelonephritis (ICD-9:590; ICD-10:N10). Septic arthritis (ICD-9: 711A, 730A; ICD-10:M00, M01.0, M46.2-3, M46.5, M86.0). Endocarditis (ICD-9:421; ICD-10: I33). CNS infection (ICD-9:320, 324; ICD-10:A39.0, G00-01, G06).

## Competing interests

Gunnar Engström was formerly employed as a senior epidemiologist by AstraZeneca R & D.

## Authors’ contributions

AE, MI, CGL, BL and GE participated in study design. AE, MI and AR performed data collection. MI, AR and GE performed statistical analysis. MI and AE wrote the manuscript draft. All authors read and approved the final version of the manuscript.

## Pre-publication history

The pre-publication history for this paper can be accessed here:

http://www.biomedcentral.com/1471-2334/14/163/prepub
